# Sjögren’s Syndrome Associated Dry Eye: Impact on Daily Living and Adherence to Therapy

**DOI:** 10.3390/jcm11102809

**Published:** 2022-05-16

**Authors:** Evan Michaelov, Caroline McKenna, Pierre Ibrahim, Manav Nayeni, Arpit Dang, Rookaya Mather

**Affiliations:** 1 Department of Ophthalmology, Schulich School of Medicine and Dentistry, London, ON N6A 5C1, Canada; emichaelov2018@meds.uwo.ca (E.M.); rookaya.mather@sjhc.london.on.ca (R.M.); 2 Schulich School of Medicine and Dentistry, London, ON N6A 5C1, Canada; 3 Western University, London, ON N6A 3K7, Canada; pibrahi4@uwo.ca (P.I.); mnayeni@uwo.ca (M.N.); adang8@uwo.ca (A.D.)

**Keywords:** Sjögren’s syndrome, dry eye disease, patient experience, patient adherence

## Abstract

Sjögren’s syndrome-related dry eye disease (SS-DED) often involves more severe dry eye symptoms than people with non-SS dry eye disease (DED). This cross-sectional study employed an anonymous self-administered questionnaire to understand the experience of people living with SS-DED and to identify factors affecting adherence to DED self-care. Participants reported difficulty with visual tasks such as driving, and diminished enjoyment in daily activities due to DED symptoms. Almost 80% reported being worried about a reduced quality of life due to DED, and over 50% reported fear of blindness. The most common reasons for non-adherence were cost of therapy and forgetting to instill drops. Drop rationing to reduce cost of therapy was endorsed by 83% of respondents. Only 3% of respondents had private insurance for non-prescription agents required to treat DED. A quarter of respondents reported they would not disclose non-adherence to their eye care provider. Multiple regression analysis revealed age was a significant contributor to missing drops. This is the first study to report on the financial burden experienced by SS-DED patients in Canada. This paper identified strategies used by patients to reduce the cost of therapy and its impact on adherence to treatment. Patients may be reluctant to disclose challenges regarding adherence to DED therapy, as well as fears of worsening quality of life.

## 1. Introduction

Sjögren’s syndrome (SS) is a chronic autoimmune condition that affects over 60 per 100,000 people globally [1]. Sjögren’s syndrome-related dry eye disease (SS-DED) often involves more severe dry eye symptoms than those in people with non-SS dry eye disease (DED) [2,3]. Symptoms include ocular irritation, vision disturbances and difficulty performing visual tasks [4]. Moreover, DED is associated with significant disease-related burdens including reduced quality of life as well as depression and anxiety [5,6]. The management of this chronic, non-curable disease requires active patient engagement in a self-care management plan. Self-care in DED involves daily, proactive instillation of ocular lubricants, topical anti-inflammatory agents, intake of dietary supplements as well as environmental and behavioral modifications [7].

Because DED self-care requires significant patient commitment and perseverance, it is important for clinicians to have an understanding of the patient experience living with DED. This includes physical and mental health factors, financial and social factors, and health perceptions and beliefs held by patients. All of these factors can influence a patient’s ability to engage in optimal DED self-management [8].

This study employed a self-administered questionnaire to examine the factors that may influence adherence to DED therapy and to evaluate some aspects of the patient experience living with DED. Specifically, this study explored the impact of DED on capacity to engage in daily life, including capacity to engage in dry eye self-management, as well as patient worries and anxieties associated with dry eye disease.

## 2. Materials and Methods

### 2.1. Survey Design

This study used a cross-sectional survey of patients with SS. The survey and all associated materials were compliant with the Declaration of Helsinki and approved by the Research Ethics Board of Western Ontario (REB #109667). The survey was created at an eighth-grade reading level and consisted of 26 questions in a multiple-choice format. In addition, questions regarding insurance coverage and adherence to therapy allowed free-text responses (Appendix A). The online electronic version of the survey was created using Qualtrics software (Qualtrics, Provo, UT, USA). Both the printed version and electronic version of the survey contained the same questions.

### 2.2. Survey Population

Data were collected from March to August 2018. Eligible study participants were at least 18 years of age. A previous diagnosis of Sjögren’s syndrome made by a physician or dentist was required. The electronic version of the survey was distributed to members of the Sjögren’s Society of Canada (Southwestern Ontario chapter) e-mail list via a monthly newsletter. Paper versions of the survey were administered to patients with Sjögren’s syndrome-related dry eye disease (SS-DED) who were not recipients of the Sjogren’s Society of Canada monthly email newsletter (patients of RM, cornea specialist). All surveys were completed anonymously and on a voluntary basis. Respondents were informed of the purpose of the survey and consent to participate was implied upon completion of the survey.

### 2.3. Data Analysis

Only responses from fully completed surveys were used for data analysis. Results were presented as a percentage of total responses collected for each question. Percentages were compared using Chi square tests. To identify the most significant reasons for patient non-adherence to DED therapy, multiple regression analysis was conducted. The dependent variable was defined as missing less than 25%, 25–50%, or greater than 50% of prescribed drops. The independent variables were gender, age, income tier, out-of-pocket expenses, experienced side effects from the drops, and reported difficulty with drop application. SPSS Statistics (SPSS v23.0, IBM, Armonk, NY, USA) was used to conduct data analysis, and results were considered statistically significant if *p* < 0.05.

## 3. Results

### 3.1. Demographic Information

A total of 330 surveys were submitted, of which 252 were fully completed. Eight surveys were removed as they did not have a diagnosis of SS made by a physician or dentist. The final cohort included 244 survey responses (Table 1). The majority of respondents (171, 70.1%) were diagnosed by a rheumatologist, with 183 (75.0%) diagnosed by serology testing. Fifty-one respondents were male, 183 were female, one identified as non-binary/third gender, and one preferred not to disclose. The median age of respondents was 61 years (IQR 53–68 years).

### 3.2. Dry Eye Symptoms Impacting Daily Activities

**Figure 1:**
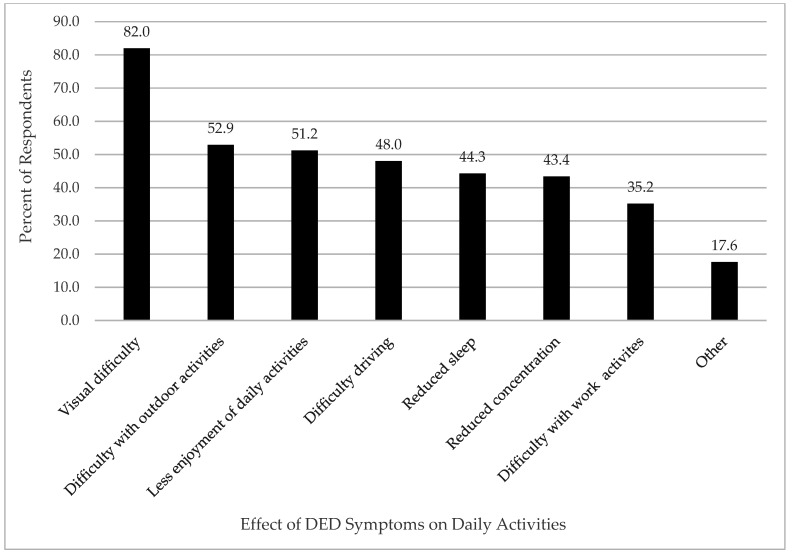
Effect of DED symptoms on daily activities.

Eighty-two percent of respondents reported difficulty with vision (Figure 1). Respondents reported difficulty with performing outdoor activities (52.9%), reading (51.2%), driving (48.0%), activities involving concentration (43.4%) and work-related activities (35.2%) due dry eye symptoms. Forty-four percent of respondents reported that dry eye symptoms interfered with sleep, and half of respondents reported diminished enjoyment in daily activities.

Participants reported the following symptoms when doses of artificial tears were missed: ocular irritation (76.6%), burning sensation (56.1%), foreign body sensation (54.5%), and itchiness (45.9%). The reported side effects from instilling prescription cyclosporine 0.05% drops included burning (30.7%), eye irritation (27.5%), and blurred vision (27.9%).

### 3.3. Emotional Well-Being and DED

Almost 80% of respondents (187; 76.6%) reported a fear of reduced quality of life in the future due to DED. More than half of respondents (52.9%) reported a fear of going blind. More than a third of respondents (39.8%) reported anxiety regarding ongoing ocular pain. Only 13 respondents (5.3%) expressed no worries about their DED.

### 3.4. Income Distribution

Income data were reported by 86.5% of respondents. Among these responses, 31.8% reported a household income < CAD 40,000 (USD 29,893), 27.5% reported a household income between CAD 40,000 and 70,000 (USD 52,315), and 19.4% reported a household income between CAD 70,000 and 100,000 (USD 74,742). A household income of >CAD 100,000 was reported by 21.8% of respondents (Table 1).

### 3.5. Private Insurance

Nearly half (43.9%) of respondents did not have any form of private insurance. Moreover, 35.7% of respondents reported having private medical insurance that covered the cost of topical cyclosporine 0.05%, which was the only on-label prescription medication indicated for the treatment of DED at the time of this study. Only 2.9% of respondents reported having private insurance coverage for non-prescription agents such as artificial tears.

### 3.6. Adherence to Treatment

**Figure 2:**
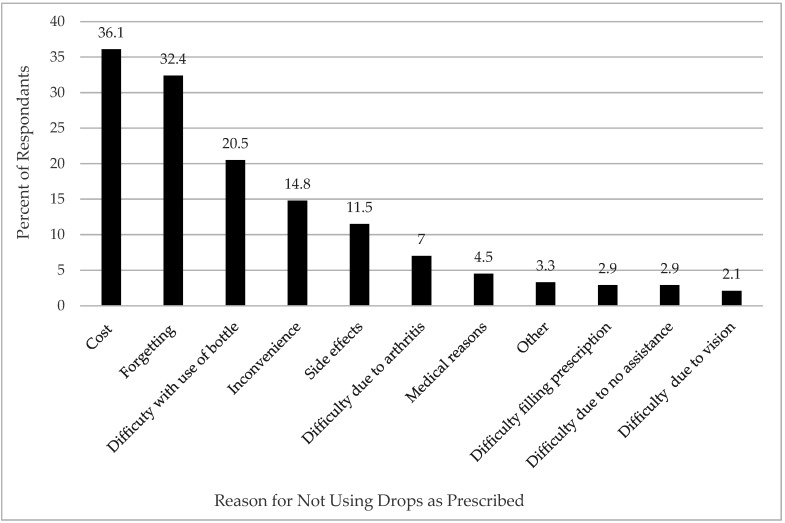
Reasons respondents not using medications as prescribed.

Cost of therapy was the most commonly reported reason (36.1%) for missing doses of artificial tears and prescription drops. Other reasons reported were: forgetting to instill doses (32.4%), difficulty administering drops from the bottles (20.5%), inconvenience (14.8%), and side effects from drops (11.5%) (Figure 2). Examples of free-text responses from participants relating to non-adherence are presented in Appendix B, question 15.

### 3.7. Variables Influencing Missed Drops

Multiple regression analysis for percentage of missed drops was performed with the following independent variables: gender, age, income tier, out-of-pocket expenses, side effects from drops, and reported difficulty with the instillation of drops. The overall model fit was acceptable with *p* < 0.01. However, the pseudo-R square revealed an inadequate level of variance (Nagelkerke R^2^ = 0.16). Of the individual variables, only age was statistically significant (*p* = 0.04). However, the contributions were minimal, with a log proportional odds estimate of −0.03 (SE 0.01) per year. This represents an odds estimate of decreasing non-adherence by approximately 3% for every year of age, if all analyzed variables are held constant.

### 3.8. Cost-Saving Strategies

**Figure 3:**
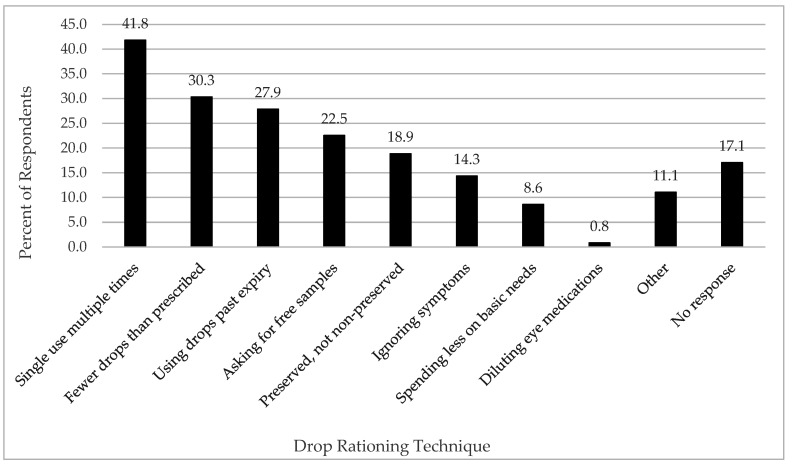
Drop-rationing techniques used by respondents.

Drop-rationing employed to reduce the cost of therapy was reported by 82.9% of respondents. Drop rationing strategies included using single-use vials more than once, which was reported by 41.8% of respondents. Other strategies included using drops past the expiry date (27.9%), using fewer drops per day than prescribed (30.3%), and requesting free samples from eye care providers (22.5%) (Figure 3). Of the 213 patients who were advised to use preservative-free artificial tear drops, 84.5% were using a drop-rationing technique to reduce the financial impact of therapy. Comparatively, of the 30 patients who reported not being advised to use preservative-free drops, 73.3% were using a strategy to ration their preserved artificial tear drops (*p* = 0.19). One respondent did not disclose whether they were advised to use preserved or preservative-free artificial tears. Examples of free-text responses relating to drop rationing strategies are shown in Appendix B question 16.

### 3.9. Disclosure of Non-Adherence

**Figure 4:**
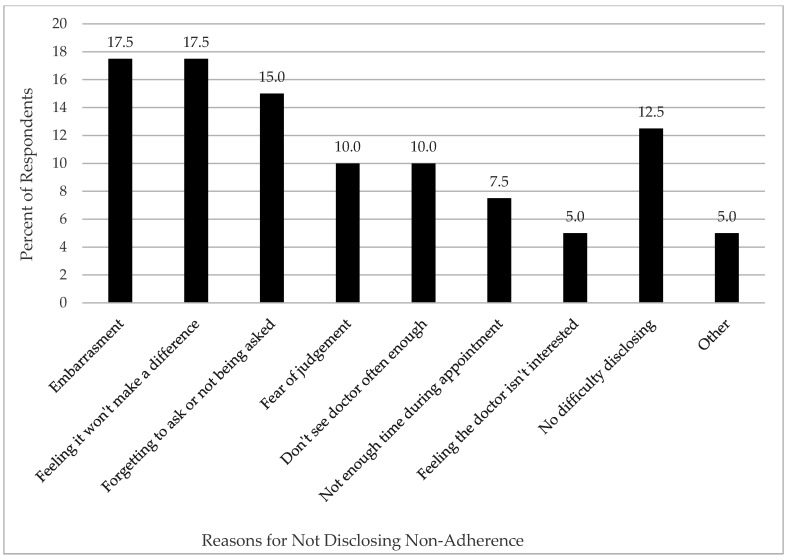
Reasons for not disclosing non-adherence to DED therapy to eye care providers.

Sixty respondents (24.7%) reported they would not disclose non-adherence to their eye care provider. Of these respondents, 40 (66.4%) reported reasons for not disclosing non-adherence (Figure 4). Reasons included embarrassment about missing drops (17.5%), with two respondents disclosing embarrassment about being unable to afford the drops; 17.5% indicated feeling that disclosing non-adherence “would not make a difference”; 15.0% reported forgetting to mention it or not being asked about adherence by the eye care provider; 10.0% reported a fear of judgment by the eye care provider; 10.0% felt they did not see their eye care provider often enough to discuss adherence; 7.5% stated not having enough time during appointments to discuss the subject of adherence; and 5.0% felt that the eye care provider was not interested in hearing about it. One respondent specifically reported not attending follow-up appointments with the eye doctor due to the associated cost.

## 4. Discussion

Dry eye disease (DED) is a common ocular condition with wide-ranging and often under-recognized functional impairments. SS-DED is often a more severe type of DED, affecting more than 430,000 people in Canada [9]. While the understanding of the pathophysiology of DED is continually evolving [10], and newer therapeutics are being developed, appreciation of the patient experience and the factors affecting patient adherence to therapy is limited. The objective of this study was to examine the experience of patients living with DED, including fears and anxieties, and capacity to engage in optimal DED self-care.

Common DED symptoms experienced by patients with SS in our study included ocular irritation, burning, foreign body sensation, and itching. In addition, effects on vision and visual tasks were identified to negatively affect quality of life and contribute to burden of disease, as reported in previous studies [4,5,6]. Specifically, respondents reported difficulty with reading, driving, work-related activities, outdoor activities, concentration and even sleep. Similarly, a recent systemic review and meta-analysis by Au et al. (2019) reported that DED may be associated with poor sleep quality and sleep duration [11].

The results of this study and those of Saldanha et al. highlight the significant emotional burden associated with living with SS [12]. In this this study, participants were asked about what worried them most about their DED. The most commonly reported concern was a *fear of reduced quality of life in the future*. The second most common anxiety for people living with SS-DED was a *fear of blindness in the future*, as reported by over half of respondents. The authors of this study consider the above responses to be highly insightful with regard to understanding the patient experience. The term patient experience encompasses an individual’s experience of their illness impacting their capacity to engage in all aspects of daily life including their perceptions of recovery, prognosis and future life [6,8]. The perceptions held by DED patients regarding their future disease course, such as blindness and poor quality of life, are important for clinicians to ask about and/or address when communicating with patients. These perceptions are likely to affect mental health and may influence self-management practices as recognized in other chronic diseases [13,14]. Patient experience is an area of DED research that warrants further study.

Several studies have identified financial barriers as having a negative impact on adherence to DED therapy [15,16]. Recent Canadian census data indicate that the median Canadian household income is CAD 70,000 [17]. In our study, 59.4% of respondents had a household income of less than the median Canadian household income, and 30% of respondents reported an annual household income of less than CAD 40,000. This is highly noteworthy because DED therapy is expensive. Non-prescription lubricants, nutritional supplements, special eyewear and prescription anti-T-cell agents are not covered by the publicly funded provincial healthcare plan in Ontario. Despite more than half of respondents reporting having private medical insurance, only 35.7% reported having coverage for prescription eye medications, and only 3% of respondents had coverage for non-prescription lubricant drops and ointments. Lack of insurance coverage for medically necessary DED treatments poses a significant financial burden, especially for patients with lower incomes.

To our knowledge, our study is the first to report on the financial burden of DED therapy in a Canadian SS-DED patient population. The average annual out-of-pocket expense was CAD 1089 in our study population. This is comparable to a study by Yu et al. (2011), which estimated that DED therapy costs the average American patient from USD 678 USD for mild DED to USD 1267 for severe DED yearly, as well as a total societal cost of over USD 11,300 per patient per year. Because patients with SS tend to have a more severe form of DED, the estimated societal cost is likely substantially higher [12]. While we recognize limited applicability of these U.S. findings when applied to other national healthcare systems, we predict that the financial burden is likely even greater in countries without government-funded healthcare insurance programs. Despite Canada’s relatively equitable healthcare insurance program, the cost of DED care still poses a significant financial burden for Canadian DED patients. Given the relatively low prevalence yet high disease burden of SS-DED, it would be advisable for provincial health insurance plans to cover some DED therapies for patients with SS.

It is clear from our study that the cost associated with DED medications motivates patients to reduce their medication-related expenses by rationing their drops. Such strategies have not been examined in the literature previously. Given the financial and insurance status of the survey population, it is not surprising that almost 83% of respondents used one or more strategies to reduce the financial burden of therapy, with a third of respondents intentionally missing doses. Common drop rationing strategies included using single-use vials multiple times, using drops past the expiry date, and using preserved rather than preservative-free artificial tears. Difficulty administering drops was another factor reported to affect adherence to therapy. Reasons may be due to blurred vision or comorbidities such as rheumatoid arthritis, peripheral neuropathy, and other SS-associated conditions [18]. Poor adherence can lead to suboptimal therapeutic benefit and loss of trust with the treatment, as discussed by Tatham et al. (2013) [19]. Other factors affecting adherence included burning, irritation and visual disturbance following instillation of drops, deterring patients from using drops as prescribed. Multiple regression analysis revealed that of the independent variables analyzed, only age had a statistically significant influence on the percentage of drops missed. Older respondents were less likely to miss drops; however, it is unclear whether this difference would make a clinically significant impact on outcomes. This finding is in keeping with that of Uchino et al., who found that patients who did not instill eyedrops regularly were more likely to be younger and have a higher rate of contact lens use and over-the-counter eye drop use [20]. On the other hand, this finding is contrasted with the literature that shows that medication compliance among the elderly is often complicated by chronic disease and high medication burden, which has been linked to poor compliance [21,22,23,24].

Finally, nearly a quarter of respondents indicated they would not reveal or discuss poor adherence to treatment with their eye care provider. The most common reasons for not disclosing adherence challenges included embarrassment and fear of judgment by the eye care provider. These findings highlight the importance of nonjudgmental, open communication between eye care providers and patients. Patient experience, including financial considerations, quality of life, emotional well-being, and communication with eye care providers influence patient expectations, patient adherence, and therapeutic outcomes. Understanding patient experiences in SS forms the foundation for developing an optimal and achievable self-care plan for each individual patient.

There are inherent limitations to this survey, which was based on a self-administered questionnaire. Although the survey was created at an eighth-grade reading level with mainly multiple-choice responses, this may have limited the depth of information obtained from respondents. Furthermore, for patients diagnosed with serology, the markers used to make this diagnosis were not known. It is possible that respondents may not have found a suitable response in the multiple-choice options and/or may not have provided their own response as free text. The survey lacked any verification methods to ensure that the respondents understood the questions and the research team understood the responses. In addition, data were collected prior to the COVID-19 pandemic, and patients’ tolerance of symptoms and adherence to treatment may have changed during the pandemic.

Although this study reveals several novel findings for the Sjögren’s population in Southwestern Ontario, it may not be representative of the broader Sjögren’s patient population. Most of the survey respondents were members of the Sjögren’s Society of Canada who received the electronic newsletter. There is potential bias in sample selection, as the survey included those with access to a computer or cell phone and internet, and those who could afford the Sjögren’s Society of Canada’s membership fee. Such patients could have a better capacity to engage in self-care. Future studies using a larger population size are needed to increase generalizability and expand our understanding of the patient experience in the SS-DED population.

## 5. Conclusions

This study adds to the existing literature highlighting that DED poses a substantial burden, both financially and in terms of quality of life, for patients with Sjögren’s syndrome. To our knowledge, this is the first study to report on the financial burden experienced by SS-DED patients in Canada, and the first paper to explore strategies used by patients to reduce the cost of therapy and its impact on adherence to treatment in Canada. It is important for eye care providers to appreciate the impact of DED symptoms, socioeconomic status, and emotional challenges on the overall patient experience, including the capacity to engage in effective self-care. We also reported on the reluctance of patients to disclose challenges regarding adherence to DED therapy and the significant mental health burden related to the fear of blindness and fear of losing quality of life in patients with SS-DED. As we have emphasized, DED self-care requires the full participation of patients, which is only achievable through patient education, access to support resources and advocacy for expanded insurance coverage for patients with SS-DED.

## Figures and Tables

**Table 1:** Respondent demographics.

Total Respondents Analyzed	244
Gender (%)	
Male	59 (24.2%)
Female	183 (75.0%)
Non-binary/Third gender	1 (0.4%)
Prefer not to disclose	1 (0.4%)
Age	
Mean (SD)	59.9 (12.4)
Median (IQR 1–3)	61 (53–68)
Years since Sjögren’s Diagnosis	
Mean (SD)	9.6 (8.6)
Median (IQR1–3)	8 (3–13)
Diagnosis By (%) *:	
Ophthalmologist	58 (23.8%)
Rheumatologist	171 (70.1%)
Family Physician	54 (22.1%)
Dentist	30 (12.3%)
Private Medical Insurance	137 (56.1%)
Coverage prescription eye drops	87 (35.7%)
Coverage non-prescription eye drops	7 (2.9%)
Income Distribution (CAD)	
<$39,000	(27.5%)
$40,000–$69,999	(23.8%)
$70,000–$99,999	(16.8%)
>$100,000	(18.9%)
Prefer not to disclose	(13.5%)

* Multiple physician diagnoses were collected.

## Appendix A. Survey Questions

Patient Demographics (1) What is your year of birth? _____________
(2) How many years ago were you diagnosed with Sjögren’s syndrome? Please answer in years. _____________
(3) Who diagnosed you with Sjögren’s syndrome? - Rheumatologist (1) - Ophthalmologist (2) - Family Doctor (3) - Other Doctor (4) - Self-diagnosed (5)
(4) What testing was used to make your diagnosis? - Salivary gland biopsy (1) - Blood work (2) - Salivary flow testing (3) - None of the above (4)
Employed Therapies, Cost, and Insurance (5) Have you been advised to use preservative-free artificial tears? - Yes (1) - No (2) - Prefer not to disclose (3)
(6) How much are you spending on preservative-free artificial tears every month? - Less than $25 (1) - $25–$50 (2) - $50–$100 (3) - $100–$200 (4) - More than $200 (5)
(7) Are you currently being prescribed Restasis? - Yes (1) - No (2) - Prefer not to disclose (3)
(8) How much are you spending on your Restasis every month? - Less than $100 (1) - $100–$200 (2) - $200–$300 (3) - $300–$400 (4) - $400–$500 (5) - More than $500 (6)
(9) Do you have private medical insurance through your employer? - Yes (1) - No (2) - Prefer not to disclose (3)
(10) Does your private medical insurance cover your non-prescription artificial tears and ointments? - Yes (1) - No (2) - Prefer not to disclose (3)
(11) Does your private medical insurance cover all of your prescription eye medications? - Yes (1) - No (2) - Prefer not to disclose (3)
(12) If there is a limit to your insurance coverage, how much is your limit? __________
(13) If your insurance has a limit, what are your out-of-pocket expenses for your dry eye prescription and non-prescription tears, gels, and ointments every month?
__________
(14) If you do not have insurance, what are your out-of-pocket expenses for your dry eye prescription and non-prescription tears, gels, and ointments every month? __________
Non-Adherence and Rationing Strategies (15) What are the reasons that you may not be using your dry eye medications and artificial tears as advised by your doctor? - Cost (1) - Inconvenience (2) - Side effects (3) - Forgetting (4) - Other medical conditions (5) - Difficulty filling prescriptions (6) - Difficulty putting in drops due to vision (7) - Difficulty putting in drops due to arthritis (8) - Difficulty putting in drops due to lack of assistance (9) - Other—Please describe (10)
(16) In order to make your drops last longer, have you ever tried any of the listed techniques (select all that apply)? - Using drops past expiry (1) - Using fewer drops than prescribed (2) - Using the same single-use medications for more than one day (3) - Using preserved drops instead of non-preserved (4) - Diluting eye medications (adding other liquids to decrease cost) (5) - Ignoring symptoms to avoid cost (6) - Spending less on basic needs to buy medicine (7) - Asking for free samples from physicians (8) - Other—Please describe (9)
(17) How many times per day have you been advised to use your artificial tears? ___________
(18) How many times per day do you use your artificial tears on average? ___________
(19) It is often difficult to follow through with medication regimens. How many drops do you miss per day? - Less than a third (1/3) of drops missed (1) - Between a third (1/3) and half (1/2) of drops missed (2) - Between half (1/2) and two thirds (2/3) of drops missed (3) - More than two thirds (2/3) of drops missed (4)
(20) How do your eyes feel when you miss your artificial tear drops? - Irritated (1) - Itchy (2) - Dry (3) - Like there is something stuck in the eye (4) - Burning (5) - Painful (6) - None (7)
(21) If you have experienced side effects from your prescription eye medications, please select them below. - Tearing (1) - Postnasal drip (2) - Visual disturbances (3) - Eye irritation (4) - Itching (5) - None (6)
Nondisclosure and Patients Fears (22) Sometimes it may be difficult to follow medical advice. Do you tell your eye doctor when you are not using your prescription medications or artificial tears as directed? - Yes (1) - No (2) - Prefer not to disclose (3)
(23) Why do you not tell your doctor about these difficulties? - Financial difficulties (1) - Physical difficulties (2) - Difficulty accessing pharmacy (3) - Other (4)
(24) Does your dry eye disease affect your life in any of the following ways? - Reduced quality or amount of sleep (1) - Reduced ability to concentrate (2) - Difficulty with vision (3) L - Limitation in your ability to enjoy your daily activities (4) - Difficulty driving (5) - Difficulty enjoying outdoor activities (6) - Difficulty with work-related activities (7)
(25) What do you worry most about your dry eye disease? - Reduction in quality of life (1) - Long-term visual changes (2) - Pain (3) - Other (4)
(26) Information about income is very important to understand. Please select the answer that includes your entire household income in 2017 before taxes in Canadian dollars. - Less than $10,000 (1) - $10,000 to $19,999 (2) - $20,000 to $29,999 (3) - $30,000 to $39,999 (4) - $40,000 to $49,999 (5) - $50,000 to $59,999 (6) - $60,000 to $69,999 (7) - $70,000 to $79,999 (8) - $80,000 to $89,999 (9) - $90,000 to $99,999 (10) - $100,000 to $149,999 (11) - $150,000 or more (12) - Prefer not to disclose (13)

## Appendix B. Patient Open-Ended Response Examples—Response Are Presented Verbatim

Q15: *What are the reasons that you may not be using your dry eye medications and artificial tears as advised by your doctor?*	Q16: *In order to make your drops last longer, have you ever tried any of the listed techniques (select all that apply)?*
“I have recently stopped Restasis after 2+ years. It burned my eyes so much I decided to give myself a break. My eyes are much better now without it (4 month).”	“Use Restasis vile twice”
“cost prevents me from using the drops on a regular basis and forces me to use them prn”	“I only use them when eyes are bothering me”
“I’m grateful for the job that I currently have & that my doctors give me a discount card ... or I’m not sure HOW I would pay for my eye drops that help me to actually see.”	“my ophthalmologist gives me a discount card to help me afford the eye drops”
“I drops pur gel in the bottle are difficult to squeeze, it takes a lot of strength to get a drop out.”	“I use a single serve of restasis for two days-once per day”
“very inconvenient because only one drugstore that can make perscription drops/must keep perscription drops refridgerated/can only get 2 weeks of drops at a time due to how its made”	“Worked more hours”
“I’m compliant for now, but cost may become an issue”	
